# Balancing risks and benefits of artificial intelligence in the health sector

**DOI:** 10.2471/BLT.20.253823

**Published:** 2020-04-01

**Authors:** Kenneth Goodman, Diana Zandi, Andreas Reis, Effy Vayena

**Affiliations:** a Institute for Bioethics and Health Policy, University of Miami, POB 016960 (M-825), Miami 33101, Florida, United States of America.; b Integrated Health Services, World Health Organization, Geneva, Switzerland.; c Research for Health, World Health Organization, Geneva, Switzerland.; d Institute of Translational Medicine, Eidgenössische Technische Hochschule Zürich, Zürich, Switzerland.

During the last decade, enhanced computing power and the availability of large amounts of data have prompted the practical use of artificial intelligence in health care. Health and medical journals now commonly include reports on machine learning and big data, and descriptions of the risks posed by, and the governance required to manage, this technology. Machine learning algorithms are used to make diagnoses, identify treatments and analyse public health threats, and these systems can learn and improve continuously in response to new data.

The tension between risks and concerns on one hand versus potential and opportunity on the other has shaped this issue of the *Bulletin of the World Health Organization* on the new ethical challenges of artificial intelligence in public health.

Data-driven discovery and analysis in health care can increase knowledge and efficiency as well as challenge social values related to privacy, data control and the monetization of personal information. In India, for example, the adoption of a system for assigning all citizens a unique identification number, linking it to individual health records and several health-related schemes, raises ethical, legal and social issues, and the need for an appropriate ethical framework and data governance.^1^ These issues might be particularly challenging in low- and middle-income countries.

Trust is perhaps the overarching theme of the contributions to this issue, and it is indeed one of the central values in digital health. One article explores opportunities for a human-centric ethical and regulatory environment to support the evolution of trust-based artificial intelligence with special regard to health insurance.^2^ Likewise, trust plays a role along with empathy and compassion in the humane side of care, the importance of which must be preserved in exploring the kind of health care society ought to promote.^3^ Similarly, European Union guidance might be too context-specific and as such leaves too much room for local, contextualized discretion for it to foster trustworthy artificial intelligence globally.^4^ In the context of population health research, researchers propose a post-research review model for ethics governance of research using artificial intelligence.^5^ For mobile health research in behavioural science, machine learning tools pose novel challenges for transparency, privacy, consent and the management of adverse events, all of which point to the need for consensus-based guidelines.^6^

As use of artificial intelligence systems expands, accountability for harm to patients and responsibility for their safety entail the need for human control and understanding of these systems.^7^ Other safeguards will require deliberate investments in data quality, access to care and processes to minimize bias, all in the service of trustworthiness.^8^ Success in integrating artificial intelligence into everyday patient care, as for instance in the United Kingdom of Great Britain and Northern Ireland’s National Health Service, is dependent on transparency, accountability and trust.^9^

In addition to trust, the values of fairness, justice and equity are seen as posing challenges even if other ethical duties are met. If artificial intelligence systems can explicitly improve equity, it is also a requirement that they do not worsen inequity.^10^ Thus, the case of neglected tropical diseases in low-resource settings illustrates opportunities for improved public health, as well as new challenges.^11^ Globally, the potential to help address some shortages and unmet needs in public health and care services might be realized by artificial intelligence-controlled conversational agents or chatbots that give health advice. However, realizing this potential will require the collaborative establishment of best practices and international ethics guidelines for technologies that replace humans.^12^

The field of bioethics emerged and grew in response to the development of new technologies and, sometimes, related wrongdoing. Ensuring adequate education, governance and ongoing ethical scrutiny will be essential if we are to realize the benefits and minimize the risks of this new technology.

Questions of artificial intelligence accountability, equity and inclusiveness remain. The field is quickly evolving, and more artificial intelligence-based applications and services are becoming available in high-income countries. Identifying better tools for benefit-sharing and, simultaneously, evidence-based safeguards and criteria for appropriate uses and users to benefit everyone, including those in middle- and low-income countries, is essential.

The World Health Organization (WHO) has made a commitment to addressing ethics, governance and regulation of artificial intelligence for health. In late 2019, WHO established an expert group to help develop a global framework for ethics and governance in artificial intelligence. The goal of this initiative is to ensure that these technologies are aligned with the overarching aims of promoting fair and equitable global health, meeting human rights standards and supporting Member States’ commitments to achieve universal health coverage.

## References

[1] Gopichandran V, Ganeshkumar P, Dash S, Ramasamy A. Ethical challenges of digital health technologies. Bull World Health Organ. 2020 4 1;98(4):277–281. 10.2471/BLT.19.237123 PMC7133485 32284652

[2] Ho CWL, Ali J, Caals K, Wagner AK. Trust-Based Ethics in the Use of AI and Big Data Analytics for Cost Prediction in Health Insurance. Bull World Health Organ. 2020 4 1;98(4):263–269. 10.2471/BLT.19.234732 PMC7133481 32284650

[3] Kerasidou A. Artificial intelligence and the ongoing need for empathy, compassion and trust in healthcare. Bull World Health Organ. 2020 4 1;98(4):245–250. 10.2471/BLT.19.237198 PMC7133472 32284647

[4] Bærøe K, Miyata-Sturm A, Henden E. Trustworthy Artificial Intelligence for Health: A Call for Global Cooperation and Targeted, Sectorial Ethical Guidance. Bull World Health Org. 2020 4 1;98(4):257–262. 10.2471/BLT.19.237289 PMC7133476 32284649

[5] Samuel G, Derrick G. Defining ethical standards for the application of digital tools to population health research. Bull World Health Organ. 2020 4 1;98(4):239–244. 10.2471/BLT.19.237370 PMC7133469 32284646

[6] Jacobson NC, Bentley KH, Walton A, Wang SB, Fortgang RG, Millner AJ, et al. Ethical dilemmas posed by mobile health and machine learning in psychiatry research. Bull World Health Organ. 2020 4 1;98(4):270–276. 10.2471/BLT.19.237107 PMC7133483 32284651

[7] Samuel G, Derrick G. Defining ethical standards for the application of digital tools to population health research. Bull World Health Organ. 2020 4 1;98(4):239–244. 10.2471/BLT.19.237370 PMC7133469 32284646

[8] Habli I, Lawton T, Porter Z. AI in Health care: Moral Accountability and Safety Assurance. Bull World Health Organ. 2020 4 1;98(4):251–256. 10.2471/BLT.19.237487 PMC7133468 32284648

[9] Paul AK, Schaefer M. The real safeguard for artificial intelligence and machine learning in global health: trustworthy health systems. Bull World Health Organ. 2020 4 1;98(4):282–284. 10.2471/BLT.19.237099 PMC7133486 32284653

[10] Smith MJ, Axler R, Bean S, Rudzicz F, Shaw J. Four equity considerations for AI in public health. Bull World Health Organ. 2020 4 1;98(4):290–292. 10.2471/BLT.19.237503 PMC7133473 32284656

[11] Vaisman A, Linder N, Lundin J, Orchanian-Cheff A, Coulibaly JT, Ephraim RKD, et al. Artificial intelligence for the image-based diagnosis of neglected tropical diseases: ethical considerations for low-resource settings. Bull World Health Organ. 2020 4 1;98(4):288–289. 10.2471/BLT.19.237560 PMC7133484 32284655

[12] Luxton DD. Ethical Challenges of Conversational Agents in Global Public Health. Bull World Health Organ. 2020 4 1;98(4):285–287. 10.2471/BLT.19.237636 PMC7133471 32284654

